# Photometric Stereo-Based Defect Detection System for Steel Components Manufacturing Using a Deep Segmentation Network

**DOI:** 10.3390/s22030882

**Published:** 2022-01-24

**Authors:** Fátima A. Saiz, Iñigo Barandiaran, Ander Arbelaiz, Manuel Graña

**Affiliations:** 1 Vicomtech Foundation, Basque Research and Technology Alliance (BRTA), Mikeletegi 57, 20009 Donostia-San Sebastián, Spain; ibarandiaran@vicomtech.org (I.B.); aarbelaiz@vicomtech.org (A.A.); 2 Computational Intelligence Group, Computer Science Faculty, University of the Basque Country, UPV/EHU, 20018 Donostia-San Sebastián, Spain; manuel.grana@ehu.es

**Keywords:** photometric stereo, quality control, deep learning, image processing, semantic segmentation

## Abstract

This paper presents an automatic system for the quality control of metallic components using a photometric stereo-based sensor and a customized semantic segmentation network. This system is designed based on interoperable modules, and allows capturing the knowledge of the operators to apply it later in automatic defect detection. A salient contribution is the compact representation of the surface information achieved by combining photometric stereo images into a RGB image that is fed to a convolutional segmentation network trained for surface defect detection. We demonstrate the advantage of this compact surface imaging representation over the use of each photometric imaging source of information in isolation. An empirical analysis of the performance of the segmentation network on imaging samples of materials with diverse surface reflectance properties is carried out, achieving Dice performance index values above 0.83 in all cases. The results support the potential of photometric stereo in conjunction with our semantic segmentation network.

## 1. Introduction

Quality control is a centerpiece of any manufacturing industry, regardless of the industrial sector. This complex and demanding process must be carried out with a high degree of precision and rigour. Moreover, the quality of manufactured components directly impacts the positioning and profit of companies in their industrial sector. Component quality inspection requires checking many aspects of the products such as its dimensions, colour or surface characteristics. Many of these inspections are usually carried out by qualified operators, especially those aspects related to surface or cosmetic defects. The main problems with this manual inspection methodology are subjectivity, monotony and being prone to human error [1]. These problems, and recent advances in computer vision and machine learning, are encouraging the trend towards the integration of automated inspection, which eliminates subjectivity and analyzes all components quickly and effectively.

Surface inspection systems are especially difficult to automatize when surfaces are highly reflective or specular because of strong image variations due to reflection. These variations tends to generate very bright image regions and very deep shadows, impeding the detection of small surface defects. This paper describes our design for a photometric stereo image acquisition technique along with a convolutional segmentation network that achieves accurate detect surface detection in highly reflective surfaces.

### 1.1. Related Works

The first neural networks appeared thanks to the ideas about unsupervised learning published by [2,3]. Subsequently, the supervised and unsupervised learning concepts were published [4] and the first neural networks were born, which were no more than variants of linear regressors. Deep neural networks were introduced by [5], who published the first learning algorithm for supervised deep feedforward multilayer perceptrons. Since that time, the development of deep learning has continued advancing, accompanied by technological progresses. These advances and the explosion of data have allowed the development of multiple applications in many different sectors, such us medicine [6], security systems [7] or robotics [8].

In the last decade there have been multiple advances in the field of machine learning. Especially, these advances come from the development of deep learning-related techniques. Deep learning architectures have become popular thanks to the increase of computing power and the availability of huge amounts of data [9,10].

Deep Learning algorithms are also increasingly applied in machine vision systems for industrial quality control. Due to the wide variety of surfaces that can be found in the manufacturing environment the way to acquire component images in each application must be customized to obtain the best image quality. Material surfaces may be specular, so choosing the right lighting schema for the acquisition of optimal images can be a difficult task. There are many works that try to eliminate the brightness produced by incident light over shiny surfaces that usually saturates image sensor regions, thus limiting any image processing in those areas [11]. For example, [12] proposes a method for specular reflection removal in a single image at the level of the individual pixel. The chromaticity of diffuse reflection is approximately estimated by employing the concept of modified specular-free image and the specular component is adjusted according to the criterion of smooth color transition along the boundary of diffuse and specular regions. Experimental results indicate that the proposed method is promising when compared with other state-of-the-art techniques, in both separation accuracy and running speed.

However, the best approach for obtaining useful images of specular surfaces is to have a reliable acquisition system that avoids surface reflections. Photometric stereo systems have been very suitable for acquiring these kind of images [13,14]. This technique estimates the object surface normals by imaging it under different lighting conditions [15]. Photometric stereo images can be used as input to a deep learning model for better defect classification. For example, a commonly used approach for classification tasks is convolutional neural networks (CNN). There are different industrial applications that combine photometric stereo acquisition and CNNs applied to defect detection. For example [16] applies this method in order to find rail defects. By means of differently colored light-sources illuminating the rail surfaces from different and constant directions, cavities are made visible in a photometric dark-field setup. Then, they experimented with classical CNNs trained in purely supervised way and also explored the impact of regularization methods, such as unsupervised layer-wise pre-training and training data-set augmentation.

In the detection of defects in the manufacturing industry, it is often also interesting to know the geometry of the defect in order to estimate its size and classify it based on criteria established by the client. For this purpose, defect detection is usually carried out by means of pixel-level segmentation. For example, [17] uses a UNet architecture for the localisation and segmentation of defects in metallic components, achieving a Dice value of 0.9167 [18]. Alternatively, [19] also propose a precise pixel-level segmentation of surface defects, using segmentation networks with different modules that allow a fast and accurate segmentation.

### 1.2. Main Contributions

Related work shows the ability of convolutional neural networks for automatic defect detection tasks but also the importance of the image quality, which is highly related with having an adequate acquisition set up according to the characteristics of the surface to be inspected. Specially in specular surfaces, topographic information is very relevant to defect characterization, thus, being a valuable source of information for its detection. Additionally, processing time is critical in order to cope with the inspection of components in high-production-rate scenarios. Therefore, the challenges that our proposed method must overcome are the following:

1. Obtain high resolution images of non-Lambertian surfaces without light reflections.
2. Use both topographic and spectral (color) information for surface analysis and defect detection.
3. Obtain a robust neural model able to detect all defects without obtaining a high false rejection rate.
4. Obtain a neural model with low processing time.

In order to overcome these challenges, we propose a system based on diffuse illumination over non-Lambertian surfaces with a white light source for extracting topographic surface information and spectral (color) response at the same time. We propose combining these sources of information into a compact RGB image format for feeding a segmentation CNN for surface defect detection. We demonstrate the benefit of this combination compared with the use of each source of information individually. We integrated the convolutional segmentation network and the photometric stereo optical sensor in an automatic inspection system for product quality assurance in a manufacturing process of metallic pieces with highly specular surface finishing and high production rates.

The structure of this paper is as follows: Section 2 describes our approach for automatic metallic component inspection based on photometric stereo images and a convolutional neural network. Section 3 describes the results obtained during the evaluation of the proposed approach. Section 4 describes the system implementation and an analysis of its performance. Subsequently, a discussion is presented in Section 5. Finally, conclusions are depicted in Section 6.

## 2. Materials and Methods

**Figure 1:**
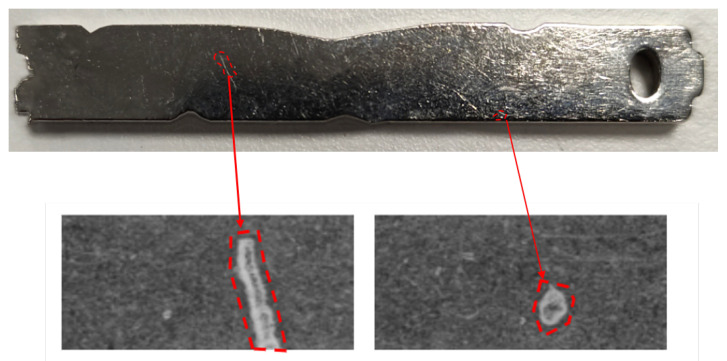
(**Top**) Conventional (non-photometric stereo) image of a manufactured component. (**Bottom**) Samples of component defects acquired with photometric stereo imaging.

The manufactured components under analysis in this work are machined nickel-plated components that may show different types of small defects on their surfaces. These components have a dimension of 50 mm × 7 mm and a thickness of 2 mm. Some samples of these defects are shown in Figure 1.

The nature of the defects is caused by the customer’s manufacturing process. This process performs some cuttings and machining on the component causing surface defects. The sizes and types of defects to be detected are established by the customer’s quality control experts.

These components, due to nickel-plated process and some coatings, have very high specular indexes resulting in very shiny components. In order to obtain high resolution and high quality images of components surfaces, we propose to use an approach based on photometric stereo.

### 2.1. Photometric Stereo Image Acquisition

Reflectance-based shape recovery of non-planar surfaces from several reflectance images obtained under different irradiance sources is a classic task in computer vision [15]. This type of approach determines the absolute depth of the surfaces by reconstructing the shape of the object under changing illumination conditions such as color or orientation, among others. This problem is called shape from shading when just one irradiance image is used for reconstruction process [20]. Photometric stereo methods firstly recover surface orientations and can be combined with an integration method to calculate a height or depth map. Even without a subsequent integration step the surface orientations can be used, for example, to determine the curvature parameters of object surfaces [21]. To acquire images for photometric stereo the object is consecutively illuminated by several light sources. In our approach we use four different light sources from four different orientations around the component to be inspected.

In this way, we get five different photometric images for each acquisition:

- Curvature images: Provides the contour lines of the surface topography.
- Texture images: Provides color or spectral response.
- Gradient image X: Signal variation in x direction.
- Gradient image Y: Signal variation in y direction.
- Range images: Computed as the image gradient magnitude. It highlights information about the changes in the intensity of the image.

To achieve these images, a system of equations introduced by Woodham [15] formalizes the solution of the problem assuming Lambertian reflectance, i.e., that the distant light sources are known point sources. Given a known vector ${\vec{I}}^{\;}$ of i observed intensities, the known matrix of normalised light directions $\left[{\vec{L}}^{\;}\right]={\left(L^{1},L^{2},L^{3}\right)}^{T}$ and the reflectivity ρ, the unknown surface normal ${\vec{n}}^{\;}$ can be obtained by inverting the following lineal equation:

(1) $${\vec{I}}^{\;}=\rho \left[{\vec{L}}^{\;}\right]{\vec{n}}^{\;}$$

If the three illumination vectors $L^{k}$ do not lie in the same plane, the matrix $\left[{\vec{L}}^{\;}\right]$ is non-singular and can be inverted, giving the following equation:

(2) $${\left[{\vec{L}}^{\;}\right]}^{-1}{\vec{I}}^{\;}=\rho {\vec{n}}^{\;}$$

As ${\vec{n}}^{\;}$ has a unit length, we can estimate the surface normal and the albedo. The problem comes when we have more than three input images; in this case the illuminations matrix $\left[{\vec{L}}^{\;}\right]$ would no longer be square, and therefore could not be inverted [22].

When the light sources are more than three, this problem is solved by least squares, using the Moore–Penrose pseudo-inverse [23] in Equation (1), thus obtaining the following solution:

(3) $${\left[{\vec{L}}^{\;}\right]}^{T}{\vec{I}}^{\;}={\left[{\vec{L}}^{\;}\right]}^{T}\left[{\vec{L}}^{\;}\right]\rho {\vec{n}}^{\;}$$

(4) $$\rho {\vec{n}}^{\;}={\left({\left[{\vec{L}}^{\;}\right]}^{T}\left[{\vec{L}}^{\;}\right]\right)}^{-1}{\left[{\vec{L}}^{\;}\right]}^{T}{\vec{I}}^{\;}$$

where ${\left({\left[{\vec{L}}^{\;}\right]}^{T}\left[{\vec{L}}^{\;}\right]\right)}^{-1}{\left[{\vec{L}}^{\;}\right]}^{T}$ is the Moore–Penrose pseudo-inverse. After that, ρ and ${\vec{n}}^{\;}$ can be solved as before.

### 2.2. Defect Segmentation Model

The proposed model is a deep network, trained with a pixel-wise loss, that considers each pixel as an individual training sample that achieves an accurate defect segmentation, adapted to its shape. With this approach we also reduce the size of the training dataset from approaches based on sliding windows. Another goal that the network has to achieve is to be able to learn the contours and engravings of each component, which are different for each product reference.

**Figure 2:**
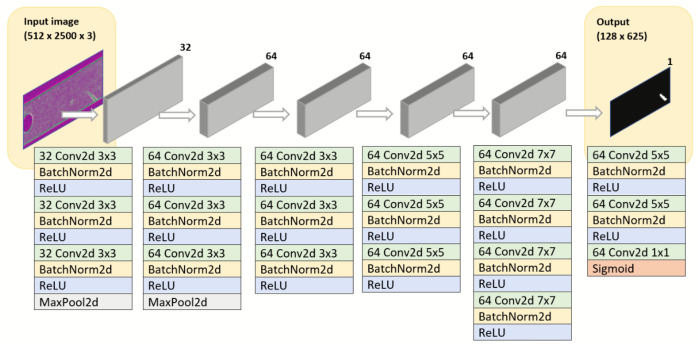
Defect segmentation network architecture.

The architecture is shown in Figure 2. The proposed network consists of six sequential layers, each composed by a combination of convolutional, normalization and max pooling operations, with an image input size of 512 × 2500. In total, the network has 19 convolutional layers for feature extraction and 2 max-pooling layers for down-sampling feature maps, combined with batch normalization and ReLU layers, in order to improve the speed, performance and stability of the network. The kernel sizes are different for each layer. In the last layers, the size is fixed and not reduced in order to have a more accurate segmentation. With this approach, we have the speed of a simple architecture with the segmentation resolution of a complex one.

This architecture is optimized for the segmentation of small defects that appears in the components.

## 3. Results

### 3.1. Data Set Generation

**Figure 3:**
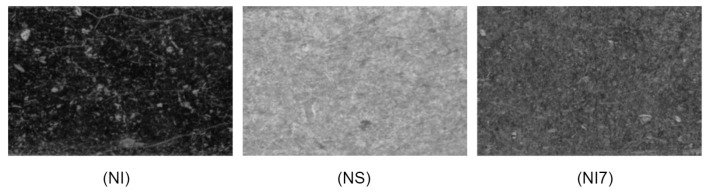
Zoom of a component region showing different texture image appearance caused by different coatings: nickel (NI), nickel–silver (NS) and Ni7 nickel–silver (Ni7).

The images acquired in this application are images of metal components with different coatings: nickel (NI), nickel–silver (NS) and Ni7 nickel–silver (Ni7). Depending on the coating’s material, the light reflected to the camera may vary, resulting in different light intensity and color detected by the sensor. Therefore, the acquisition of each material looks different and must be treated individually to obtain optimal results, thus resulting in three different sub-datasets. An example of the differences in the surface appearance is shown in Figure 3.

Our data set contains the samples shown in Table 1. The images were acquired and annotated by the quality experts of the manufacturing company.

In order to obtain a model robust to small changes that may occur during the acquisition step, we applied a data augmentation process. These type of techniques are very powerful to artificially create variations of existing images or samples by applying geometric and photometric transformations [24]. These transformations have to be adapted to the specific use case.

In our use case, we apply photometric transformations to simulate small changes on the surface coating that components may have. A change in the surface coating supposes a different response in the incident light and, therefore, differences in the acquired image brightness. Although we separate the classes into different subgroups, variations of this type may occur within each class.

We also apply geometric transformations such as horizontal and vertical flipping and image rotations of some degrees. In our case, the component is always placed horizontally, so we apply only ±2 degrees in-plane rotation in order to resemble the real manufacturing conditions.

To evaluate the trained models, new samples of the three materials were captured as described in Table 2. These testing samples were intended to be composed with equal proportions of defective and non-defective samples.

### 3.2. Performance Criteria

In the field of surface defect detection, the following statistics are often used to evaluate the results obtained:

- True Positives (*TP*): the defect is detected as a defect.
- True Negatives (*TN*): the background is detected as background.
- False Positives (*FP*): the background is mistakenly detected as a defect.
- False Negatives (*FN*): the defect is mistakenly detected as background.

With the values of the statistics described above, the following values commonly used to evaluate the quality of the segmentation can be calculated [25]:

- Mean Dice Value: is an spatial overlap-based metric. It focuses on measuring the similarity between two samples through the union and intersection of sets of predicted and ground truth pixels. This index takes values between 0 and 1, and is better when it is closer to 1, since this means having more surface in common between the ground truth and the result of the segmentation. This coefficient is calculated by the Equation (5), where A and B are the ground truth mask and the predicted mask, respectively.
  (5) $$Dice\left(A,B\right)=\frac{2∥A⋂B∥}{∥A∥+∥B∥}$$
- Sensitivity: is the ability of the model in not marking a negative sample as positive. It is measured by the formula of Equation (6).
  (6) $$Sensitivity=\frac{TP}{\left(TP+FN\right)}$$
- Specificity: is the ability to find all positive samples. It is measured by the formula of Equation (7).
  (7) $$Specificity=\frac{TN}{\left(TN+FP\right)}$$
- Pixel accuracy: the percent of pixels in the image that are classified correctly. It is calculated by the Equation (8).
  (8) $$Accuracy=\frac{TP+TN}{\left(TP+TN+FP+FN\right)}$$

In our use case, the criterion for positive detection is a Dice index greater than 0.5. The results shown in the following tables were obtained using this criteria.

### 3.3. Combination of Photometrtic Stereo Images for Defect Detection

One of our goals is to exploit the full potential of photometric stereo images. We propose creating RGB images by embedding different photometric stereo images in each color channel.

In order to compose the best combination of images, a study of their individual usefulness for defect prediction was performed. For this purpose, we trained the customized segmentation model in each image independently, obtaining the segmentation accuracy in each case, using the same NI dataset. Based on the results, the three channels that obtained the best results were texture, range and curvature as shown in Table 3.

**Figure 4:**
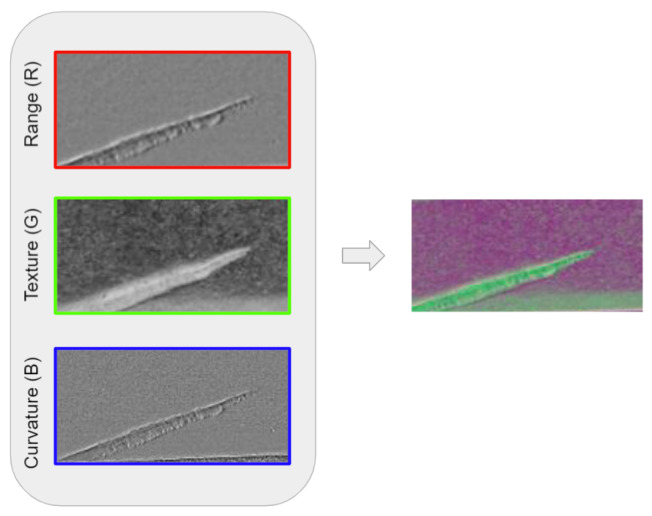
RGB image with the selected layers of photometric stereo acquisition.

We think that these three images contain better information for defect representation than gradient X and gradient Y. Given these results, we propose combining texture, range and curvature images into an RGB image for training the segmentation network, as shown in Figure 4.

### 3.4. Comparison of Segmentation Results between RGB and Texture-Only Images

The defect segmentation network of each material was trained using two different types of datasets: a dataset with a single channel images containing, only texture information, and a dataset with RGB images composed of all the photometric stereo information, i.e., with texture, range and curvature images combined. The single-channel images can resemble the images acquired by a conventional non-photometric diffuse illumination dome. With this experiment, we evaluated the potential benefits of using photometric-based imaging, comparing it with a more conventional non-photometric based approach.

As shown the results in Table 4, Table 5 and Table 6, respectively, all materials demonstrate an improvement in all metrics of the use of the RGB representation over the use of texture-only images. With the use of image combination, the false positive rate is reduced to approximately half for all materials. In addition, an increase in the rate of true positives is observed. These values are especially relevant in the industrial manufacturing environment. With respect to the Dice value, an increase of 9%, 6% and 4% is observed for NI, NS and NI7 respectively.

### 3.5. Defect Segmentation Performance for Each Material Using RGB Images

We evaluated the performance of the defect segmentation network using the RGB images composed by texture, curvature and range in each material. As shown in Table 7, the models reach a Dice value higher than 0.82, reaching a value of up to 0.94 in NI7. Regarding the performance of the algorithm in the inspection line, the results obtained on the test set reveal the good performance of the model. The achieved sensitivity and specificity ensure the defect detection without increasing the false rejection rate.

### 3.6. Comparison against Two Benchmark Segmentation Networks

To test the performance of our proposed network, we compared our approach with two well-known segmentation networks: DFANet [26] and UNet [27].

DFANet is an efficient segmentation architecture designed to be agile and to run with the minimum necessary resources. It is based on multi-scale feature propagation, thus reducing the number of parameters. Its design is intended to achieve a good trade off between speed and performance in segmentation.

We also use UNet, which is a network designed to be trained end-to-end with few image samples. In addition, thanks to its design for the medical field, it allows obtaining a very accurate detection result.

The results show that our customized segmentation network achieves better results in defect segmentation of the components, as shown in Table 8. DFANet obtains a higher number of false positives because its structure detects impurities on the non-defective surfaces that are not considered real defects. UNet obtains better results than DFANet, but fails in the detection of the defects, with only dimensional affection. If a defect does not show relevance in the texture image, this network is not able to detect it correctly, thus causing false negatives.

## 4. System Implementation

**Figure 5:**
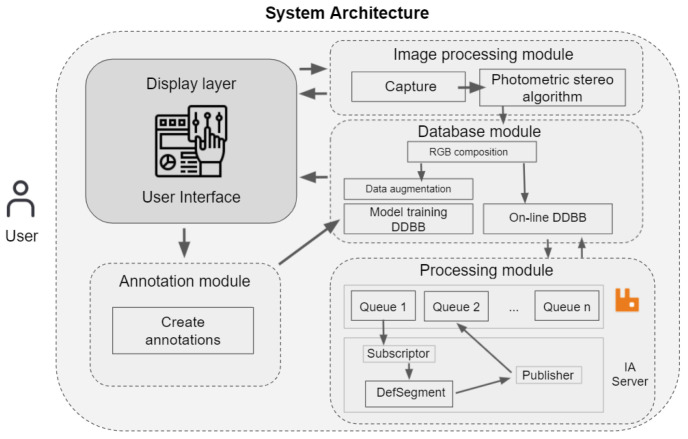
System architecture for defect detection in steel components.

Once the feasibility of the defect detection algorithms and the suitability of the acquisition system had been verified, the whole system architecture was designed and developed. Overall, the system was composed of several interoperable modules such as, image acquisition, image annotation and image processing in a distributed manner. Figure 5 shows an overview/scheme of the relation between the developed modules.

### 4.1. Acquisition Set Up

**Figure 6:**
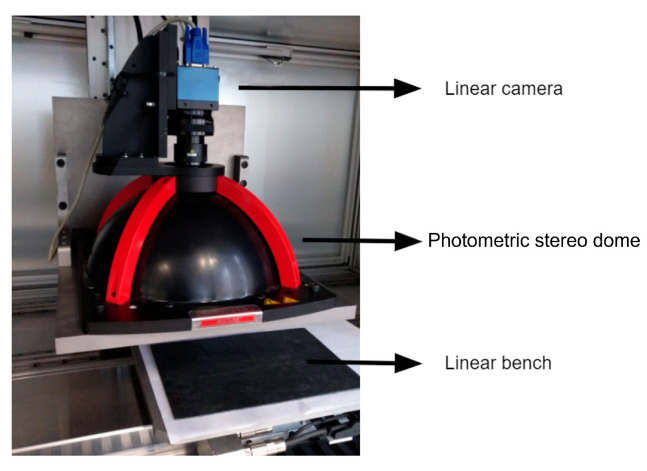
Acquisition set up to capture photometric stereo images.

For image acquisition, we combine a photometric stereo diffuse dome and a high resolution linear sensor with a telecentric lens. The field of view was chosen based on the maximum dimension of the component to be analyzed, in our case, 200 mm. To allow the inspection of small defects with enough detail (<1 mm), each line capture of the components is acquired at 4 K. Therefore, we obtain a very high-resolution capture of the component surface. This set up is shown in Figure 6.

### 4.2. Annotation Module

**Figure 7:**
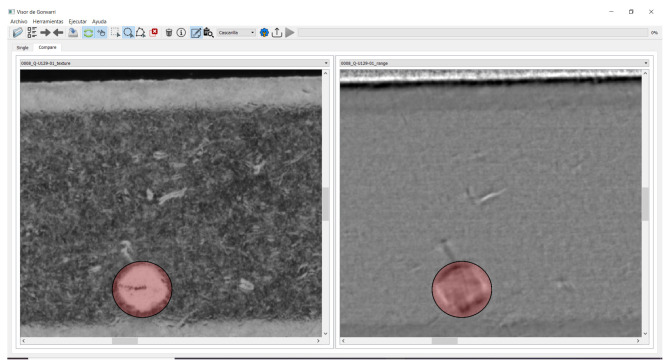
Customized annotation tool showing a side by side view of texture channel (**left**) and range channel (**right**) of an interest region of the component.

This module provides an annotation tool for capturing the knowledge of quality inspection experts. As previously described in Section 2.1, we acquire five input images during the photometric stereo surface reconstruction. In order to take advantage of this information we have developed a viewer that allows visualizing the different image layers synchronously. All types of defects are not always visible in the five photometric stereo images. For example, defects that are dimensionally affected, such as scratches, are more visible in the range and curvature images. In contrast, defects without topographic involvement, such as oxide, are more visible in the texture images. The main advantage of our customized annotation tool is that the user can visualize and interact with one, two or three channels of the same image independently. A shown in Figure 7, it allows the quality expert to discern both type of defects at the same time in a side by side view of different photometric stereo images of the same component.

### 4.3. System Performance

Besides the accuracy of the segmentation network, another fundamental point for the integration of any application in the industry is the processing time of the inspection analysis. It is mandatory that the time spent in this analysis to be under a certain cycle time, in order to allow the continuous production of the components. In this regard, a comparison of the processing execution time between an industrial computer without a GPU and an external server with a GPU is made. The external server has an NVIDIA RTX 2070, allowing performing the inference step faster than a CPU.

Our system uses a message broker as a middleware to orchestrate and communicate all the processes involved in a distributed manner. This tool facilitates the definition of exchanges and communication channels between the different modules and processes with the use of message queues. In this case, the acquisition and inspection processes are performed in different computers with dedicated hardware for each task. Thus, we can leverage the use of specialized hardware resources such as GPUs for the inference computation for our network and easily scale when required.

Performance evaluation is measured for the following processes: inference and persistence (storing the result in the database). The time breakdown for each process is shown in Table 9. In summary, the total processing time executed locally in a single PC with a CPU is 707ms. In contrast, executed in a distributed manner on the external server with dedicated GPU, the processing time is 138ms. If the application is executed locally, the processing time does not reach the required production rate, so it is necessary to distribute the processing task into an external server. This action implies a five-fold reduction of the execution time.

## 5. Discussion

As demonstrated by experimentation and results shown in previous sections, the use of a photometric stereo sensor is adequate for the application of defect detection on reflective surfaces. The proposed method of stacking multiple sources of data from the photometric stereo sensor into a high-resolution, multi-channel (RGB) image supposes an improvement in the accuracy and performance achieved with the presented convolutional neural network. A typical problem with this type of application is the detection of very small defective regions with respect to the dimension of the component. This makes it difficult to avoid false positives, as the dataset is unbalanced between the amount of normal and defect pixels. Therefore, the extra information provided by the multiple data layers from the photometric stereo allows the model to extract features related with defective regions in a more optimal way.

Another challenge to overcome in machine vision applications developed for industrial manufacturing processes is the speed at which they must operate to satisfy high production rates. Defect segmentation applications are often not fast enough to be integrated into an industrial production line. However, our proposed system is able to process images in the required cycle time. We demonstrate that, if the inference is performed on an industrial computer (CPU), the required time is not achieved. Hence, a distributed computation for parallelizing processes is mandatory. This is also a benefit in terms of organization and scalability, since the same system can inspect components in several production lines and more hardware resources can be easily added to cope with the required compute demand.

In comparison to other inspection methods proposed in the state of the art, our method allows the inspection of reflective surfaces in the cycle time required by the manufacturing industry. Other methods propose multiple image acquisitions from different angles to avoid surface reflections, thus affecting the acquisition time and also the required space for the installation of the inspection station in the production line. Compared with the methods that employ deep learning, our method is novel in using multiple combined sources of information, allowing a more detailed and robust description of the component surface. This feature gives greater stability in defect detection compared with traditional image acquisition-based systems. In addition, a specific segmentation network is created, allowing the exploitation of this information in a fast and accurate way. The proposed acquisition system can be also used on non-planar geometries, such as cylindrical components, just by modifying the automation of the component’s displacement. The proposed method has disadvantageous in terms of hardware costs and the level of automation required for image acquisition. A photometric stereo dome is electronically more complex than conventional illumination, supposing a cost penalty. Furthermore, automation is needed to move the component in a constant and controlled way, which must be perfectly synchronised with the trigger of the camera and the dome.

## 6. Conclusions

This work proposes an automatic system for the quality inspection of metallic components using a photometric stereo based sensor and a customised segmentation model. We propose combining the photometric stereo information that better resemble the defects. For this purpose, a comparison analysis was carried out for each photometric stereo image, which allows choosing the most suitable image combination.

The photometric stereo image combination provides additional information to the segmentation network increasing the effectiveness and accuracy of the defect detection. This approach is confirmed in different materials showing a decrease in false rejection rate and an increase in the true detection rate.

A performance comparison with different well-known segmentation architectures was carried out to demonstrate the suitability of our proposed segmentation network.

Finally, some performance tests were carried out in terms of execution time. These tests demonstrate the need of accelerated hardware and parallel processing capabilities, such as, making use of distributed computing and GPU devices to fulfill the required speed in the production line.

## Figures and Tables

**Table 1:** Number of acquired training images for each coating and each defect type.

Number of Samples in the Training Dataset			
Coating Type	Non-Defective Samples	Bump Marks and Scratches Samples	Total Images
nickel (NI)	427	464	891
nickel–silver (NS)	489	470	959
Ni7 nickel–silver (Ni7)	465	434	899

**Table 2:** Number of test images for each coating.

Number of Samples in the Testing Dataset	
Coating Type	Samples
nickel (NI)	297
nickel–silver (NS)	417
Ni7 nickel–silver (Ni7)	320

**Table 3:** Defect segmentation results for each photometric stereo image using our customized segmentation network.

Segmentation Results for Each Photometric Stereo Image in NI Dataset	
Image Type	Accuracy
texture	0.9259
range	0.9482
curvature	0.9326
gradient X	0.7865
gradient Y	0.8103

**Table 4:** Nickel material segmentation results.

Value	NI (RGB)	NI (Texture Only)
Dice mean	0.8957	0.8027
sensitivity	0.98	0.9477
specificity	0.931	0.9027
accuracy	0.956	0.9259
TP	150	145
TN	134	130
FP	10	14
FN	3	8

**Table 5:** Nickel Silver material segmentation results.

Value	NS (RGB)	NS (Texture Only)
Dice mean	0.829	0.767
sensitivity	0.954	0.9081
specificity	0.883	0.819
accuracy	0.916	0.8609
TP	186	178
TN	196	181
FP	26	40
FN	9	18

**Table 6:** Ni7 material segmentation results.

Value	NI7 (RGB)	NI7 (Texture Only)
Dice Mean	0.9739	0.9619
sensitivity	0.9855	0.9710
specificity	0.9615	0.9505
accuracy	0.9690	0.9593
TP	136	134
TN	175	173
FP	7	9
FN	2	4

**Table 7:** Material Segmentation results.

Value	NI	NS	NI7
Dice Mean	0.8957	0.829	0.9739
sensitivity	0.98	0.954	0.9855
specificity	0.931	0.883	0.9615
accuracy	0.956	0.916	0.9690
TP	150	186	136
TN	134	196	175
FP	10	26	7
FN	3	9	2

**Table 8:** Defect segmentation results using DFANet and UNet on NI dataset.

Segmentation Results Using NI Dataset	
Neural Network	Accuracy
DFANet	0.8732
UNet	0.9113
Our network	0.9560

**Table 9:** Processing times.

Action	Time CPU (ms)	Time GPU (ms)
inference time	471	126
DDBB storage	236	12
total time (ms)	707	138

## Data Availability

Not available.
